# Brain-derived neurotrophic factor promotes VEGF-C-dependent lymphangiogenesis by suppressing miR-624-3p in human chondrosarcoma cells

**DOI:** 10.1038/cddis.2017.354

**Published:** 2017-08-03

**Authors:** Chih-Yang Lin, Shih-Wei Wang, Yen-Ling Chen, Wen-Yi Chou, Ting-Yi Lin, Wei-Cheng Chen, Chen-Yu Yang, Shih-Chia Liu, Chia-Chu Hsieh, Yi-Chin Fong, Po-Chuan Wang, Chih-Hsin Tang

**Affiliations:** 1 Graduate Institute of Basic Medical Science, China Medical University, Taichung, Taiwan; 2 Department of Medicine, Mackay Medical College, New Taipei City, Taiwan; 3 Department of Fragrance and Cosmetic Science, College of Pharmacy, Kaohsiung Medical University, Kaohsiung, Taiwan; 4 Department of Orthopedic Surgery, Kaohsiung Chang Gung Memorial Hospital Medical Center, Kaohsiung, Taiwan; 5 Department of Orthopaedics, MacKay Memorial Hospital, Taipei, Taiwan; 6 Institute of Biomedical Engineering and Nanomedicine, National Health Research Institutes, Miaoli County, Taiwan; 7 Institute of Molecular Medicine, National Tsing-Hua University, Hsinchu, Taiwan; 8 Department of Sports Medicine, College of Health Care, China Medical University, Taichung, Taiwan; 9 Department of Orthopaedic Surgery, China Medical University Beigang Hospital, Yun-Lin County, Taiwan; 10 Department of Gastroenterology, Hsinchu MacKay Memorial Hospital, Hsinchu City, Taiwan; 11 Department of Biotechnology, College of Health Science, Asia University, Taichung, Taiwan; 12 Department of Pharmacology, School of Medicine, China Medical University, Taichung, Taiwan

## Abstract

Chondrosarcoma is the second most common primary malignancy of bone, and one of the most difficult bone tumors to diagnose and treat. It is well known that increased levels of vascular endothelial growth factor-C (VEGF-C) promote active tumor lymphangiogenesis and lymphatic tumor spread to regional lymph nodes. Brain-derived neurotrophic factor (BDNF) is known to promote metastasis in human chondrosarcoma cells. Knowing more about the mechanism of BDNF in VEGF-C expression and lymphangiogenesis in human chondrosarcoma would improve our understanding as how to prevent chondrosarcoma angiogenesis and metastasis, which currently lacks effective adjuvant treatment. Here, we found that BDNF expression was at least 2.5-fold higher in the highly migratory JJ012(S10) cell line as compared with the primordial cell line (JJ012). In addition, VEGF-C expression and secretion was markedly increased in JJ012(S10) cells. Conditioned medium from JJ012(S10) cells significantly promoted migration and tube formation of human lymphatic endothelial cells (LECs), whereas knockdown of BDNF attenuated LEC migration and tube formation by suppressing VEGF-C production in JJ012(S10) cells. Mechanistic investigations indicated that BDNF facilitated VEGF-C-dependent lymphangiogenesis through the MEK/ERK/mTOR signaling pathway. We also showed that microRNA (miR)-624-3p expression was negatively regulated by BDNF via the MEK/ERK/mTOR cascade. Importantly, BDNF knockdown profoundly inhibited tumor-associated lymphangiogenesis *in vivo*. Further analyses identified that BDNF promoted tumor lymphangiogenesis by downregulating miR-624-3p in human chondrosarcoma tissues. In conclusion, this study is the first to reveal the mechanism underlying BDNF-induced lymphangiogenesis. We suggest that BDNF may serve as a promising therapeutic target for the restriction of VEGF-C-mediated tumor lymphangiogenesis and lymphatic metastasis.

Chondrosarcoma is a malignant tumor composed of cartilage-producing cells. It is the second most common primary malignancy of bone and one of the most difficult bone tumors to diagnose and treat. In general, chondrosarcoma occurs most commonly in adults aged between 40 and 60 year-old, and primarily involves the pelvis, the sternum, the ribs and the scapula. The primary treatment for chondrosarcoma is surgical resection, and the response to chemotherapy or radiation is poor. High recurrence rates and metastatic propensity pose major obstacles for current treatment of chondrosarcoma.^1^ Thus, a greater understanding of the molecular mechanisms influencing metastasis in chondrosarcoma will help us to develop novel therapeutic strategies to impede the progression of this disease.

The growth and formation of new lymphatic vessels (lymphangiogenesis), is for a crucial step in the metastatic spread of cancer cells, first to the sentinel lymph nodes surrounding the tumor and then throughout the body via the lymphatic system. Vascular endothelial growth factor-C (VEGF-C) makes a crucial contribution to lymphangiogenesis and metastasis.^2, 3^ VEGF-C is predominantly expressed and released by tumor cells, and activates the proliferation, migration, and tube formation of human lymphatic endothelial cells (LECs) via VEGF receptor-3 (VEGFR-3),^4^ and that elevated VEGF-C levels are significantly correlated with lymph node metastasis and poor patient prognosis.^5^ However, despite its importance in tumor physiology and pathology, we know very little about appropriate therapeutic targets against tumor-associated lymphangiogenesis. Although our recent studies have revealed that VEGF-C is closely related to tumor stage in human chondrosarcoma,^2, 3^ we need to learn more about the mechanisms involved in VEGF-C-dependent lymphangiogenesis in order to fully understand the microenvironment in chondrosarcoma microenvironment.

MicroRNAs (miRNAs) are small non-coding RNAs containing around 22 nucleotides that have an essential role as regulators of various pathological processes, including carcinogenesis and tumor dissemination.^6^ miRNAs control gene expression by binding to the 3′-untranslated region (3′-UTR) of the messenger RNAs (mRNAs) through complementary base pairing, resulting in mRNA degradation or translation inhibition. Circulating miRNAs have been reported as serving as potential biomarkers and therapeutic targets in cancer.^7^ In addition, miRNAs are involved in various functions of the cancer cell, such as survival, proliferation, migration, invasion and angiogenesis.^8^ Accumulating evidence indicates that miRNAs suppress tumor lymphangiogenesis by inhibiting VEGF-C expression.^9^ For example, miR-128 targets VEGF-C expression in bladder cancer and thereby inhibits tumor lymphangiogenesis and metastasis,^10^ whereas miR-300 diminishes the expression and production of VEGF-C as well as tumor lymphangiogenesis in human oral cancer.^11^

Brain-derived neurotrophic factor (BDNF) is a member of the neurotrophin family.^12^ Recently, several studies have indicated that BDNF has an important role in the pathogenesis of numerous tumor types, including neuronal (neuroblastoma) and non-neuronal tumors, such as lung, gastric, breast, hepatocellular and colorectal cancer.^12, 13^ Compelling evidence indicates that BDNF is associated with tumorigenesis and metastasis in several types of cancer.^14, 15^ Our previous research has proposed that BDNF promotes chondrosarcoma metastasis through the upregulation of integrin *β*5 and matrix metalloproteinase-1 expression.^16, 17^ Moreover, we have described how BDNF induces VEGF-A expression and angiogenesis in human chondrosarcoma cells.^18^ VEGF-C is known to mediate tumor lymphangiogenesis in many human cancers.^19, 20^ However, the effect of BDNF on VEGF-C expression and lymphangiogenesis in chondrosarcoma has not been well explored. In this study, we investigated the role of BDNF in VEGF-C-dependent lymphangiogenesis, to elucidate its mechanism of action in human chondrosarcoma cells.

## Results

### Knockdown of BDNF decreases VEGF-C expression and lymphangiogenesis

**Figure 1:**
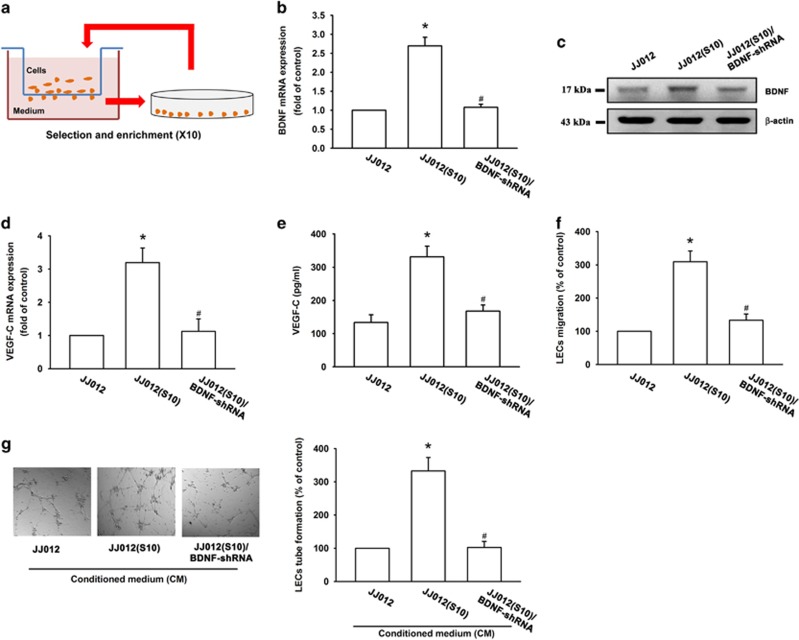
Endogenous BDNF of chondrosarcoma regulates VEGF-C expression and lymphangiogenesis. (**a**) Establishment of high migration chondrosarcoma cell lines. (**b**-**e**) BDNF and VEGF-C expression in indicated cells was examined by RT-qPCR, ELISA and western blotting. (**f**) The CM was also applied to the lower chamber of a Transwell migration assay. LECs were applied to the upper chamber and migrated LECs were quantified. (**g**) The CM was applied to LECs and a capillary-like structure formation in LECs was examined by tube formation. Quantitative results are expressed as the mean±S.E.M. *P<0.05 as compared with the JJ012 group; ^#^*P*<0.05 as compared with the JJ012(S10) group

Our previous studies have shown that BDNF is associated with chondrosarcoma metastasis and angiogenesis.^16, 17, 18^ VEGF-C has been reported to mediate tumor lymphangiogenesis in many human cancers.^19, 20^ However, the effect of BDNF on VEGF-C expression and lymphangiogenesis in chondrosarcoma has not been well explored. We used the Transwell assay to select highly migratory JJ012(S10) cells (Figure 1a). We found that JJ012(S10) cells displayed higher BDNF and VEGF-C expression as compared with JJ012(S0) cells (Figures 1b and e). Incubation of human LECs with conditioned medium (CM) from JJ012(S10) cells markedly increased LEC migration and tube formation (Figures 1f and g). We used five BDNF short-haired (sh)RNAs to determine the function of BDNF expression by western blotting. The BDNF-2-shRNA was the most efficient at inhibiting BDNF expression (Supplementary Figure S1), so we therefore used BDNF-2-shRNA in our further experiments. We found that knockdown BDNF significantly reduced BDNF and VEGF-C expression in JJ012(S10) cells (Figures 1b and e), and subsequently abolished LEC migration and tube formation (Figures 1f and g). These data indicate that BDNF promotes lymphangiogenesis by increasing VEGF-C production in human chondrosarcoma cells. However, incubation of SW1353 and JJ012 cells with 0–30% CM from JJ012(S10) cells only slightly increased VEGF-C expression (Supplementary Figure S2). It may be that the difference in BDNF of 30% CM between JJ012(S10) with SW1353 and JJ012 cells is not enough to significantly enhance VEGF-C expression.

### Involvement of VEGF-C expression in BDNF-directed lymphangiogenesis of chondrosarcoma

**Figure 2:**
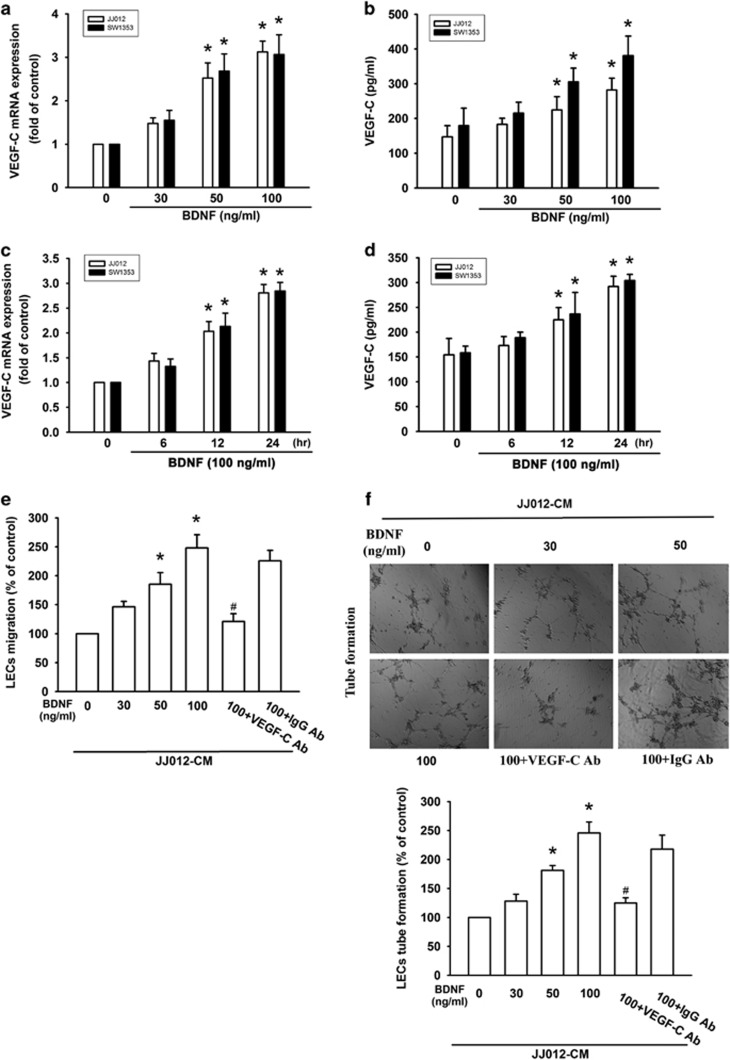
BDNF increases VEGF-C expression and lymphangiogenesis. (**a** and **b**) Chondrosarcoma cells were incubated with various concentrations of BDNF (30–100 ng/ml) for 24 h, and VEGF-C expression was examined by RT-qPCR and ELISA. (**c** and **d**) Chondrosarcoma cells were incubated with BDNF (100 ng/ml) for indicated time intervals, and VEGF-C protein and mRNA expression was examined by RT-qPCR and ELISA. (**e**) The CM was also applied to the lower chamber of a Transwell migration assay. LECs were applied to the upper chamber and migrated LECs were quantified. (**f**) The CM was applied to LECs and the capillary-like structure formation in LECs was examined by tube formation. Quantitative results are expressed as the mean±S.E.M. *P<0.05 as compared with the control group; ^#^*P*<0.05 as compared with the BDNF-treated group

To verify the role of BDNF in VEGF-C-dependent lymphangiogenesis, we directly applied BDNF to chondrosarcoma cells, and determined VEGF-C expression and secretion. As shown in Figures 2a and d, BDNF increased mRNA level and secretion of VEGF-C in a concentration- and time-dependent manner, as determined by quantitative real-time polymerase chain reaction (RT-qPCR) and ELISA assays. We also found that CM from BDNF-treated chondrosarcoma cells markedly enhanced LEC migration and tube formation (Figures 2e and f). Notably, pretreatment of CM with VEGF-C neutralizing antibody significantly suppressed BDNF-activated migration and tube formation of LECs. These data show that BDNF promotes VEGF-C-dependent lymphangiogenesis in human chondrosarcoma cells.

### MEK and ERK activation are involved in BDNF-induced VEGF-C expression and lymphangiogenesis

**Figure 3:**
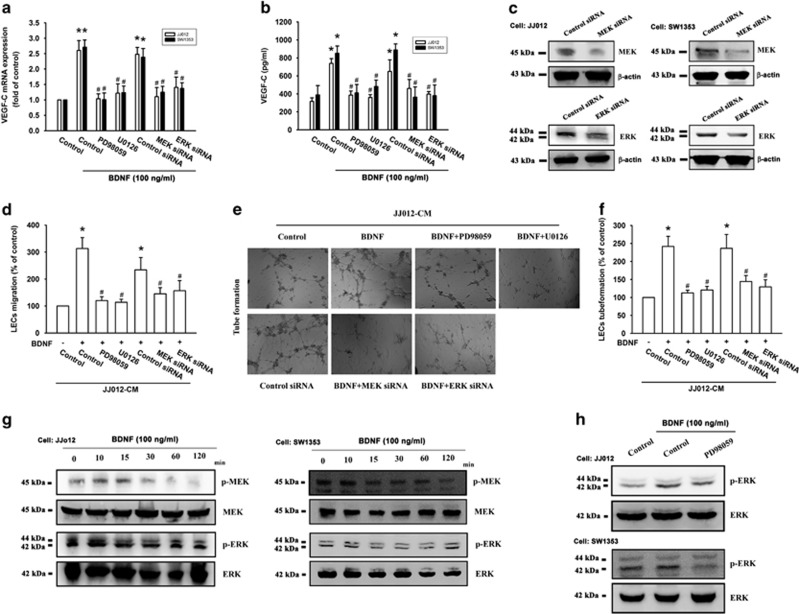
The MEK/ERK pathway mediates BDNF-induced VEGF-C production and lymphangiogenesis. Cells were pretreated with MEK or ERK inhibitors, or pretransfected with siRNAs as indicated, then stimulated with BDNF for 24 h; VEGF-C expression was measured by qPCR, western blotting and ELISA (**a**-**c**). (**d**-**f**) Medium was collected as CM, then applied to LECs and analyzed for migration activity as well as tube formation activity. (**g**) Chondrosarcoma cells treated with BDNF for the indicated times were analyzed by western blotting with MEK and ERK antibody. (**h**) Chondrosarcoma cells were pretreated with the MEK inhibitor as indicated and then incubated with BDNF for 15 min and analyzed by western blotting with ERK antibody. Quantitative results are expressed as the mean±S.E.M. *P<0.05 as compared with the control group; ^#^*P*<0.05 as compared with the BDNF-treated group

The mitogen-activated protein kinase kinase (MEK) and its downstream signaling extracellular signal-regulated kinase (ERK) have been implicated in cancer progression processes such as metastasis, angiogenesis or lymphangiogenesis.^21, 22, 23^ We therefore examined whether the MEK/ERK pathway is involved in BDNF-mediated VEGF-C expression and lymphangiogenesis. Treatment with a MEK inhibitor (PD98059) or ERK inhibitor (U0126), as well as transfection with a MEK short interfering (si)RNA or an ERK siRNA, all diminished BDNF-induced VEGF-C expression (Figures 3a and c). In addition, BDNF-induced LEC migration and tube formation were reduced by treatment with pharmacological inhibitors or siRNA transfection (Figures 3d and f). Stimulation with BDNF increased MEK and ERK phosphorylation in a time-dependent manner (Figure 3g). Incubation of cells with the MEK inhibitor antagonized BDNF-induced ERK phosphorylation (Figure 3h). These results suggest that BDNF acts through the MEK/ERK pathway to enhance VEGF-C-dependent lymphangiogenesis in human chondrosarcoma cells.

### BDNF promotes VEGF-C expression and lymphangiogenesis through the mTOR pathway

**Figure 4:**
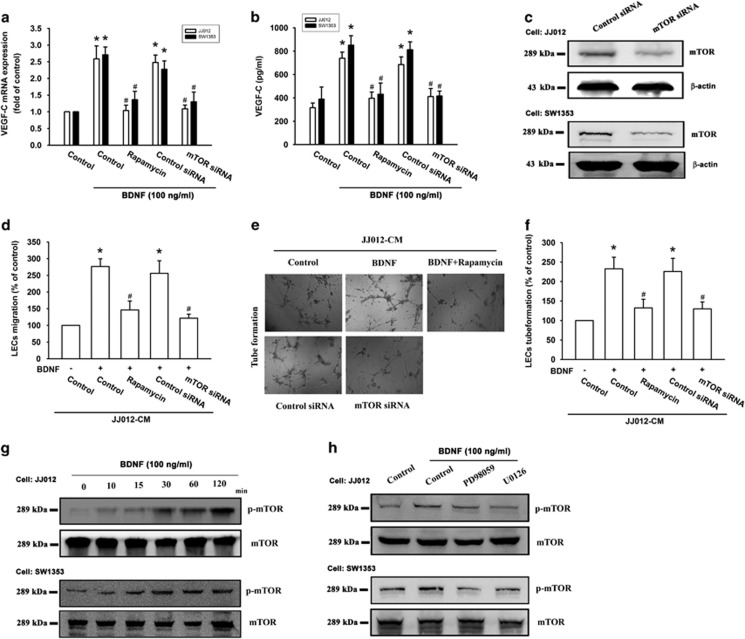
The mTOR pathway mediates BDNF-induced VEGF-C production and lymphangiogenesis. Cells were pretreated with the mTOR inhibitor or pretransfected with siRNA as indicated then stimulated with BDNF for 24 h; VEGF-C expression was measured by qPCR, western blotting and ELISA (**a**-**c**). (**d**-**f**) Cells were pretreated with a pharmacological inhibitor or pretransfected with siRNA as indicated, then stimulated with BDNF for 24 h. The CM was then applied to LECs, which were analyzed for migration activity as well as tube formation activity. (**g**) Chondrosarcoma cells treated with BDNF for the indicated times were analyzed by western blotting with the mTOR antibody. (**h**) Chondrosarcoma cells were pretreated with MEK and ERK inhibitors, as indicated, and then incubated with BDNF for 120 min and analyzed by western blotting with the mTOR antibody. Quantitative results are expressed as the mean±S.E.M. *P<0.05 as compared with the control group; ^#^*P*<0.05 as compared with the BDNF-treated group

Previous studies have shown that mTOR, located downstream of the MEK/ERK signaling pathway,^24, 25^ is associated with tumor progression, angiogenesis and lymphangiogenesis.^26^ We therefore analyzed the role of mTOR in BDNF-mediated VEGF-C expression and lymphangiogenesis. We found that an mTOR inhibitor (rapamycin) and siRNA both abolished BDNF-induced VEGF-C expression in chondrosarcoma cells (Figures 4a and c). Moreover, BDNF-induced LEC migration and tube formation were suppressed by treatment with mTOR inhibitor and siRNA (Figures 4d and f). We further showed that mTOR phosphorylation was increased after BDNF treatment in chondrosarcoma cells (Figure 4g). In addition, incubation of cells with MEK or ERK inhibitors antagonized BDNF-induced mTOR phosphorylation (Figure 4h). These data demonstrate that BDNF induces VEGF-C expression in chondrosarcoma cells, and subsequently promotes lymphangiogenesis in LECs through the MEK/ERK/mTOR pathway.

### BDNF regulates miR-624-3p directly binding to 3'-UTR of VEGF-C in human chondrosarcoma cells

**Figure 5:**
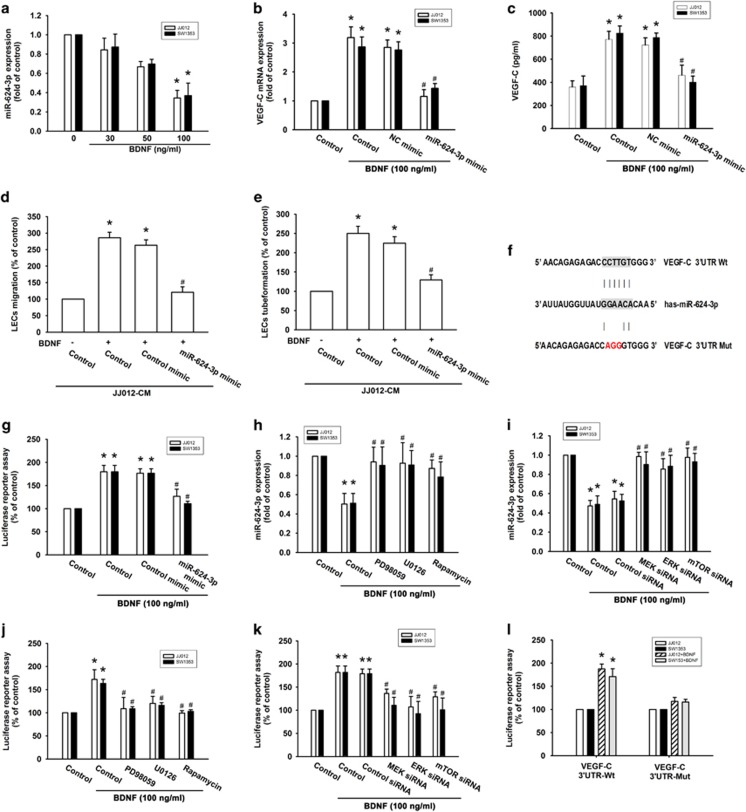
BDNF increases VEGF-C expression by suppressing miR-624-3p expression. (**a**) Cells were incubated with BDNF for 24 h and the miR-624-3p was examined by qPCR. (**b** and **c**) Cells were pretransfected with the miRNA mimic as indicated then incubated with BDNF for 24 h; VEGF-C expression was measured by RT-qPCR and ELISA. (**d** and **e**) CM was applied to LECs, which were analyzed for migration activity as well as tube formation activity. (**f**) Schematic 3′-UTR representation of human VEGF-C containing the miR-624-3p binding site. (**g**) Chondrosarcoma cells were transfected with miR-624-3p luciferase plasmids before incubation with BDNF for 24 h. Luciferase activity was assayed. (**h**-**k**) Incubation with MEK, ERK and mTOR inhibitors or siRNA reversed BDNF-mediated miR-624-3p expression and VEGF-C-3′-UTR luciferase activity. (**l**) Cells were co-transfected with VEGF-C-3′-UTR-wt or VEGF-C-3′-UTR-mut plasmid for 24 h then stimulated with BDNF, and relative luciferase activity was measured. Quantitative results are expressed as the mean±S.E.M. *P<0.05 as compared with the control group; ^#^*P*<0.05 as compared with the BDNF-treated group

Increasing evidence has suggested that miRNAs are important regulators of tumor-associated lymphangiogenesis.^27, 28^ miRNA target prediction using open-source software (TargetScan, miRDB, miRBase and RNA22) revealed that the 3′-UTR of VEGF-C mRNA harbors potential binding sites for miR-624-3p. As shown in Figure 5a, BDNF inhibited miR-624-3p expression in a concentration-dependent manner. We next examined whether miR-624-3p regulated BDNF-enhanced VEGF-C production, by transiently transfecting the miR-624-3p mimic into BDNF-treated chondrosarcoma cells. We found that miR-624-3p mimic significantly reduced BDNF-induced VEGF-C expression and secretion (Figures 5b and c). In addition, the miR-624-3p mimic markedly inhibited BDNF-induced LEC migration and tube formation (Figures 5d and e). Notably, transfection of miR-624-3p mimic also increased miR-624-3p expression (Supplementary Figure S3).

To learn whether miR-624-3p regulates the 3′-UTR region of VEGF-C, we constructed luciferase reporter vectors harboring the wild-type 3′-UTR region of VEGF-C mRNA (VEGF-C-3′-UTR-wt) and a vector containing mismatches in the predicted miR-624-3p binding site (VEGF-C-3′-UTR-mut) (Figure 5f). We found that transfection with the miR-624-3p mimic antagonized BDNF increased luciferase activity in the VEGF-C-3′-UTR-wt plasmid (Figure 5g). In addition, treatment with MEK, ERK and mTOR inhibitors or siRNA reversed BDNF-mediated miR-624-3p expression and VEGF-C-3′-UTR luciferase activity (Figures 5h and k). Moreover, the results indicated that BDNF increased luciferase activity in the VEGF-C-3′-UTR-wt plasmid but not in the VEGF-C-3′-UTR-mut plasmid (Figure 5l). Collectively, these data suggest that miR-624-3p directly represses VEGF-C expression via binding to the 3′-UTR region of the human VEGF-C gene through the MEK/ERK/mTOR pathway.

### BDNF boosts tumor-associated lymphangiogenesis *in vivo* and increases VEGF-C expression by downregulating miR-624-3p in specimens of chondrosarcoma patients

**Figure 6:**
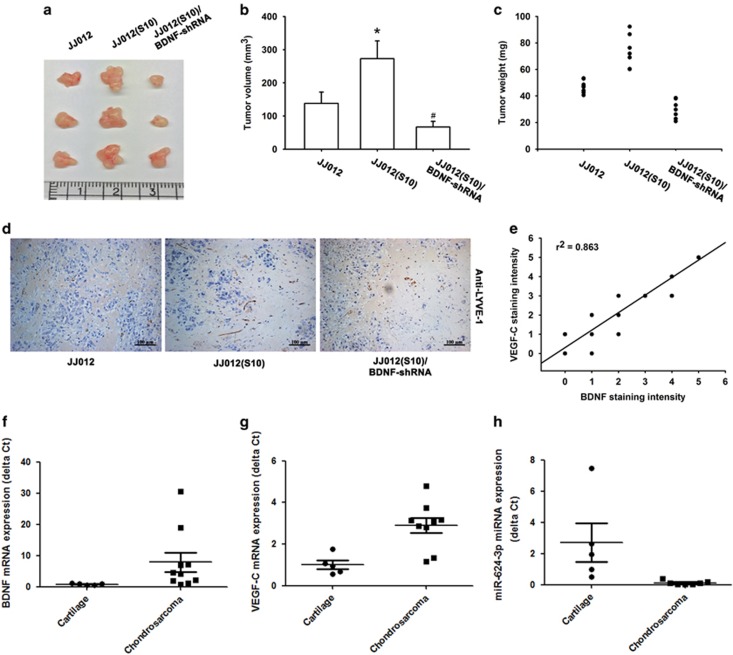
The correlation of BDNF, VEGF-C, miR-624-3p and lymphangiogenesis in clinical significance and animal model *in vivo*. (**a**-**c**) At 28 days after injection, the tumors were excised, photographed with a microscope, weighed and measured. The tumor sections were immunostained with LYVE-1 antibody (**d**). (**e**) The correlation and quantitative data of IHC of BDNF and VEGF-C expression levels in normal cartilage and chondrosarcoma patients. (**f**-**h**) The mRNA expression of BDNF, VEGF-C and miR-624-3p in normal cartilage and chondrosarcoma tissue were examined by RT-qPCR. Quantitative results are expressed as the mean±S.E.M. *P<0.05 as compared with the JJ012 group; ^#^*P*<0.05 as compared with the JJ012(S10) group

Next, we examined whether BDNF knockdown suppresses tumor-associated lymphangiogenesis *in vivo*. We used three chondrosarcoma cell lines (JJ012, JJ012(S10) and JJ012(S10)/BDNF-shRNA), each of which was mixed with Matrigel, then injected into the right flanks of SCID mice. We found that BDNF knockdown inhibited tumor growth and the expression of the lymphatic vessels marker LYVE-1 (Figure 6a and d). These results indicate that BDNF enhances tumor-associated lymphangiogenesis in a chondrosarcoma xenograft model.

In our previous studies, immunohistochemical (IHC) analyses of human chondrosarcoma tissue have shown that levels of BDNF and VEGF-C expression are highly correlated with tumor stage.^3, 18^ Here, we further demonstrated that BDNF expression was strongly correlated with VEGF-C expression in human chondrosarcoma specimens (Figure 6e). In order to confirm the clinical significance of BDNF in chondrosarcoma lymphangiogenesis, we performed RT-qPCR analysis to compare the expression profiles of BDNF and VEGF-C between normal cartilage and chondrosarcoma tissue. As shown in Figure 6f and g, BDNF and VEGF-C expression levels were higher in tumor specimens than in normal tissue. Furthermore, the data clearly showed low miR-624-3p expression in chondrosarcoma patients (Figure 6h). Our findings imply that BDNF enhances VEGF-C expression by suppressing miR-624-3p in chondrosarcoma patients.

## Discussion

Our previous studies have demonstrated that lymphangiogenesis is one of the major routes for tumor invasion and metastasis in chondrosarcoma.^3, 29^ VEGF-C is a key modulator in tumor lymphangiogenesis and metastasis, so is therefore a potential target for preventing lymphatic metastasis.^2^ Here, we provide novel insights into the role of BDNF in tumor-associated lymphangiogenesis. Our data show that BDNF promoted VEGF-C expression and increased lymphangiogenesis by downregulating miR-624-3p through the MEK/ERK/mTOR signaling pathway. In addition, we demonstrate a clinical correlation between BDNF and VEGF-C in human chondrosarcoma tissue. Tyrosine receptor kinase B (TrkB) is a major receptor of BDNF and is associated with tumor lymphangiogenesis.^30^ We found higher expression of TrkB and lower expression of miR-624-3p in JJ012(S10) cells compared with both JJ012 and SW1353 cells (Supplementary Figure S4). This pattern is similar to the clinical expression of higher BDNF and VEGF-C expression versus lower miR-624-3p expression in human chondrosarcoma tissue compared with normal cartilage. Therefore, targeting BDNF may bea novel therapeutic strategy for the treatment of chondrosarcoma.

Previous studies have indicated that the MEK/ERK pathway serves as the pivotal regulator in a variety of cell functions, including growth, proliferation, angiogenesis and lymphangiogenesis.^22, 31^ The activation of mTOR causes protein synthesis, cell survival, motility, invasion, lymphangiogenesis and differentiation, which ultimately can lead to cancer initiation and progression.^32, 33^ It has been reported that the AKT/mTOR and MEK/ERK pathways participate in cross-talk in cancer cells. For example, Ras is capable of activating the PI3K/AKT/mTOR pathway in addition to the Raf/MEK/ERK pathway, whereas ERK can activate mTOR signaling.^24^ Interestingly, the AKT/mTOR and MEK/ERK pathways are known to be activated in some sarcoma subtypes.^34, 35, 36^ However, the mechanism underlying signal transduction between MEK, ERK and mTOR signaling in chondrosarcoma remains unclear. Here, we found that MEK, ERK and mTOR inhibitors or siRNA antagonized BDNF-induced VEGF-C production, as well as the migration and tube formation of LECs. Stimulating chondrosarcoma cells with BDNF enhanced the phosphorylation of MEK, ERK and mTOR, which indicates that the MEK/ERK/mTOR cascade has a key role in BDNF-induced VEGF-C-dependent lymphangiogenesis. Furthermore, treatment of cells with a MEK inhibitor or ERK inhibitor significantly reduced BDNF-activated ERK and mTOR phosphorylation. Thus, our results show that BDNF promotes VEGF-C expression and lymphangiogenesis in human chondrosarcoma cells via the MEK/ERK/mTOR signaling pathway.

Deregulated biogenesis of miRNAs has been widely implicated in cancer progression and metastasis. Increasing evidence suggests that several miRNAs can reduce tumor progression via direct repression of VEGF-C. miR-27b, miR-101, miR-128 and miR-206 have been shown to inhibit lymphangiogenesis and metastasis in a variety of human cancer cells, via the targeting of VEGF-C.^27, 37, 38^ This current study demonstrates that BDNF markedly inhibited the expression of miR-624-3p in human chondrosarcoma cells and specimens. Transfection with the miR-624-3p mimic significantly reduced BDNF-induced VEGF-C production, and LEC migration as well as tube formation. We also found that miR-624-3p directly inhibited VEGF-C production through binding to the 3’-UTR of the human VEGF-C gene, and thereby negatively regulating VEGF-C-mediated lymphangiogenesis. These findings provide new insight into a potential miRNA-based molecular therapeutic strategy for VEGF-C-mediated lymphangiogenesis.

BDNF has been considered to be an important factor during carcinogenesis.^39^ BDNF serves as a poor prognostic factor and facilitates tumor progression, but its precise role in tumor lymphangiogenesis has largely remained unknown.^40^ In this study, the mRNA data demonstrate significantly higher BDNF and VEGF-C expression in chondrosarcoma patients compared with that in healthy individuals. Conversely, miR-624-3p expression was significantly lower in chondrosarcoma patients. *In vitro* and *in vivo* data show in this study that BDNF promotes VEGF-C expression and lymphangiogenesis by downregulating miR-624-3p expression through the MEK/ERK/mTOR signaling pathway. Thus, BDNF may be a new molecular therapeutic target in chondrosarcoma lymphangiogenesis and metastasis.

## Materials and methods

### Materials

Rabbit polyclonal antibodies specific for p-MEK and p-mTOR were purchased from Cell Signaling Technology (Danvers, MA, USA). Rabbit polyclonal antibodies specific for BDNF, MEK, p-ERK, ERK, mTOR, VEGF-C and *β*-actin were purchased from Santa Cruz Biotechnology (Santa Cruz, CA, USA). LYEV-1 antibody was purchased from Abcam (Cambridge, MA, USA). Recombinant human BDNF was purchased from R&D Systems (Minneapolis, MN, USA). ON-TARGETplus siRNAs were purchased from Dharmacon Research (Lafayette, CO, USA). The miR-624-3p mimic, miRNA control, Lipofectamine 2000, and Trizol were purchased from Life Technologies (Carlsbad, CA, USA). DMEM, *α*-MEM, fetal bovine serum and all other cell culture reagents were purchased from Gibco-BRL life technologies (Grand Island, NY, USA). The BDNF-shRNA plasmids were purchased from RNAiCore (Taipei, Taiwan); their sequences are provided in Supplementary Table S1. The pSV-*β*-galactosidase control vector and luciferase assay kit were purchased from Promega (Madison, WI, USA). All other chemicals were purchased from Sigma-Aldrich (St. Louis, MO, USA).

### Cell culture

The human chondrosarcoma cell line (JJ012) was kindly provided by Dr. Sean P Scully (University of Miami School of Medicine, Miami, FL, USA).^41^ Highly migratory JJ012(S10) cells were selected in our laboratory, according to our previous methods.^42, 43^ Subpopulations of JJ012 cells were examined by Transwell assay and selected according to their differential invasiveness. After overnight migration, cells that had penetrated through pores and migrated to the underside of filters were trypsinized and harvested for a second round of selection. After 10 rounds of selection, a migration-prone subline was designated as JJ012(S10) (Figure 1a). The human chondrosarcoma cell line (SW1353) was obtained from the American Type Culture Collection (ATCC, Manassas, VA, USA). Human telomerase-immortalized human dermal LECs (hTERT-HDLECs), an immortalized human LEC line, were purchased from Lonza (Walkersville, MD, USA). Culture conditions were recorded for all cells as detailed in our previous paper.^18, 29^

### Western blot analysis

Cellular lysates were prepared as described previously.^44^ Proteins were resolved by sodium dodecyl sulfate-polyacrylamide gel electrophoresis and transferred to immobilon polyvinyl difluoride membranes (lmmobilon P, Millipore, Billerica, MA, USA). The blots were blocked with 4% non-fat milk for 1 h at room temperature and then probed with rabbit anti-human antibodies against p-MEK, MEK, p-ERK, ERK, p-mTOR, mTOR, *β*-actin or BDNF (1:1000) for 1 h at room temperature. After undergoing three washes, the blots were incubated with goat anti-rabbit or goat anti-mouse peroxidase-conjugated secondary antibody (1:1000) for 1 h at room temperature, and subjected to a further three washes. Blots were visualized by enhanced chemiluminescence, using Imagequant LAS 4000 (GE Healthcare, Pewaukee, WI, USA).

### Quantitative real-time polymerase chain reaction

Total RNA was extracted from chondrosarcoma cells using a TRIzol kit (MDBio Inc., Taipei, Taiwan). We performed the reverse transcription reaction using 1 *μ*g of total RNA that was reverse transcribed into cDNA using the MMLV RT kit (Invitrogen, Carlsbad, CA, USA). We performed the RT-qPCR analysis using the Taqman One-Step RT-PCR Master Mix (Applied Biosystems, CA, USA). RT-qPCR analysis of miRNA expression was performed on the StepOnePlus sequence detection system, using the TaqMan MicroRNA Reverse Transcription Kit and normalized to U6 expression.^42^

### Plasmid construction and transient transfection

VEGF-C-3′-UTR-wt was constructed into the pmir-GLO reporter vector between the *Pme*I and *Xho*I cutting sites, according to the manufacturer’s instructions. To analyze 3′-UTR luciferase activity, chondrosarcoma cells were transfected with VEGF-C-3′-UTR-wt or VEGF-C-3′-UTR-mut luciferase plasmids. Cells were lysated after 24 h of transfection, harvested and detected using a luciferase assay system (Promega, Madison, WI, USA).

ON-TARGETplus siRNAs of MEK, ERK, mTOR and control were purchased from Dharmacon Research. Transient transfection of siRNAs was carried out using DharmaFECT1 transfection reagent. The siRNA (100 nM) was formulated with DharmaFECT1 transfection reagent, according to the manufacturer's instructions.

### ELISA assay

Human chondrosarcoma cells were cultured in 24-well plates then incubated in a humidified incubator at 37 °C for 24 h. To examine the downstream signaling pathways involved in BDNF treatment, cells were pretreated with various inhibitors for 30 min before the addition of BDNF (100 ng/ml). After incubation, the medium was removed and stored at –80 °C until the assay was performed. VEGF-C in the medium was assayed using a VEGF-C enzyme immunoassay kit (R&D Systems), according to the procedure described by the manufacturer.

### LEC migration assay

This process used Transwell inserts (8-*μ*m pore size; Corning, Costar, Tewksbury, MA, USA) in 24-well plates. Chondrosarcoma cells were pretreated for 30 min with designated inhibitors or vehicle (0.1% dimethyl sulfoxide) or transfected with designated siRNAs for 24 h, then incubated with BDNF for 24 h, after which time the CM was collected. LECs were seeded into the upper chamber of a Transwell assay and 300 *μ*l of CM was placed in the lower chamber. After 20 h, migrated cells were stained with crystal violet and counted under a microscope.

### LEC tube formation assay

Matrigel (BD Biosciences, Bedford, MA, USA) was dissolved overnight at 4 °C and 48-well plates were prepared with 100 *μ*l Matrigel in each well then incubated at 37 °C overnight. LECs were resuspended at a density of 2 × 10^4^/200 *μ*l in CM (50% EGM-MV2 medium and 50% chondrosarcoma cell CM) and added to the wells. After 6 h of incubation at 37 °C, LEC tube formation was assessed by microscopy, and each well was photographed. The number of tube branches and total tube lengths were calculated using MacBiophotonics Image J software (Bethesda, MD, USA).

### *In vivo* tumor xenograft study

Four-week-old male CB17-SCID mice were randomized into three groups (JJ012, JJ012(S10) or JJ012(S10)/BDNF-shRNA). Experimental cells growing exponentially were implanted into 10 SCID mice by subcutaneous injection of 2 × 10^6^ cells resuspended in 200 *μ*l of medium containing 50% serum-free DMEN/*α*-MEM and 50% Matrigel. After 28 days, the tumors were removed and fixed in 10% formalin, and their volume and weight were measured.

### Immunohistochemistry

The tissues were placed on glass slides, rehydrated and incubated in 3% hydrogen peroxide to block endogenous peroxidase activity. After trypsinization, the sections were blocked by incubation in 3% bovine serum albumin in PBS. The primary polyclonal antibodies, rabbit anti-human LYVE-1, were applied to the slides at a dilution of 1 : 200 and incubated at 4 °C overnight. After being washed three times in PBST, the samples were treated with goat anti-rabbit IgG biotin-labeled secondary antibodies at a dilution of 1 : 50. Bound antibodies were detected with an ABC kit (Vector Laboratories, Burlingame, CA, USA). The slides were stained with chromogen diaminobenzidine, washed, counterstained with Delafield's hematoxylin, dehydrated, treated with xylene and mounted.

### Patients and specimen preparation

The study protocol was approved by the Institutional Review Board of China Medical University Hospital. All patients gave written consent before enrollment. Tumor tissue specimens were collected from patients diagnosed with chondrosarcoma who underwent surgical resection at China Medical University Hospital.

### Statistical analysis

Data are presented as the mean±S.E.M. of at least three independent experiments. Statistical analysis of comparisons between two samples was performed using the Student’s *t*-test. One-way analysis of variance with Bonferroni’s *post-hoc* tests was used for statistical comparisons of more than two groups. In all cases, *P*<0.05 was considered to be statistically significant.
