# Supplementary material for: Brain-derived neurotrophic factor promotes VEGF-C-dependent lymphangiogenesis by suppressing miR-624-3p in human chondrosarcoma cells
Source: Cell Death Dis. 2017 Aug 3;8(8):e2964–. doi: 10.1038/cddis.2017.354 (PMC5596545; doi:10.1038/cddis.2017.354)
Supplement: Supplementary Data [file cddis2017354x1.doc]

**Supplementary data**

**Table S1. BDNF-shRNA sequence details and information.**

| **Gene Symbol** | **Clone ID** | **Oligo Sequence** |
| --- | --- | --- |
| BDNF-1 | TRCN0000058208 | CCGGGCTCAGTAGTCAAGTGCCTTTCTCGAGAAAGGCACTTGACTACTGAGCTTTTTG |
| BDNF-2 | TRCN0000058209 | CCGGGCAATACTTCTACGAGACCAACTCGAGTTGGTCTCGTAGAAGTATTGCTTTTTG |
| BDNF-3 | TRCN0000058210 | CCGGGCCCTTACCATGGATAGCAAACTCGAGTTTGCTATCCATGGTAAGGGCTTTTTG |
| BDNF-4 | TRCN0000058211 | CCGGGAAGCAAACATCCGAGGACAACTCGAGTTGTCCTCGGATGTTTGCTTCTTTTTG |
| BDNF-5 | TRCN0000058212 | CCGGCTGTTGGATGAGGACCAGAAACTCGAGTTTCTGGTCCTCATCCAACAGTTTTTG |


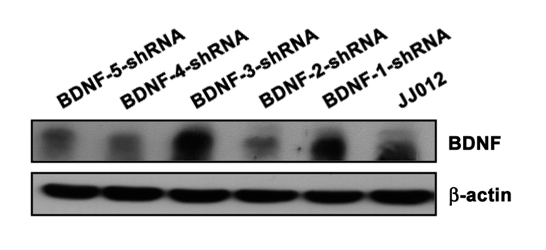


**Fig. S1. The knockdown efficiency of BDNF-shRNAs in BDNF expression.** JJ012 cells were infected with BDNF-shRNAs and the BDNF expression was subsequently examined by Western blot.


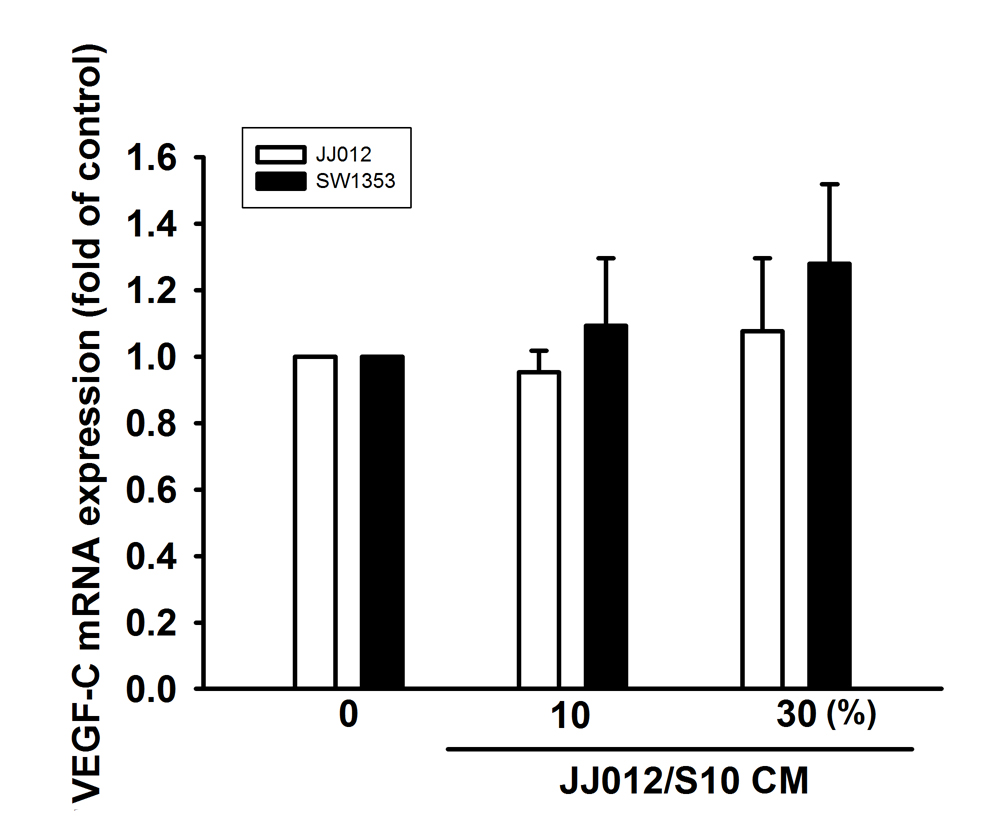


**Fig. S2.** **CM from JJ012(S10) cells only slightly increased VEGF-C mRNA expression in SW1353 and JJ012 cells.** SW1353 and JJ012 cells were treated with CM (0–30%) from JJ012/S10 cells for 24 h, and VEGF-C expression was measured by RT-qPCR. Quantitative results are expressed as the mean ± SEM.


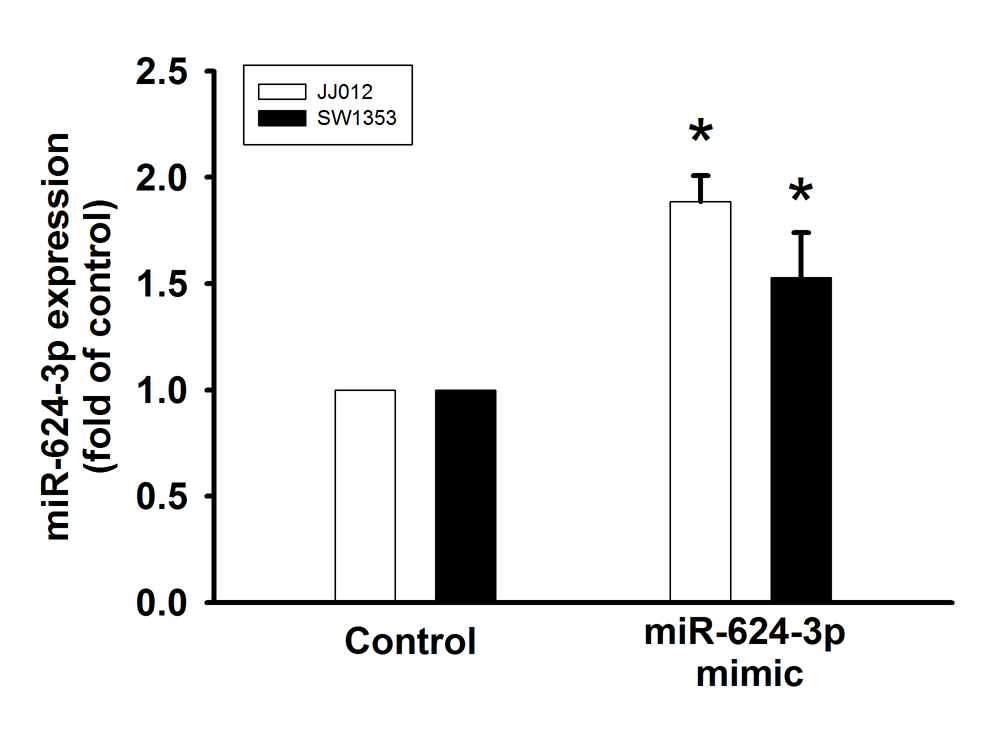


**Fig. S3. The miR-624-3p mimic increased miR-624-3p expression.** Chondrosarcoma cells were transfected with control or miR-624-3p mimic for 24 h, and miR-624-3p expression was measured by RT-qPCR. Quantitative results are expressed as the mean ± SEM. **P* < 0.05 as compared with the control group.


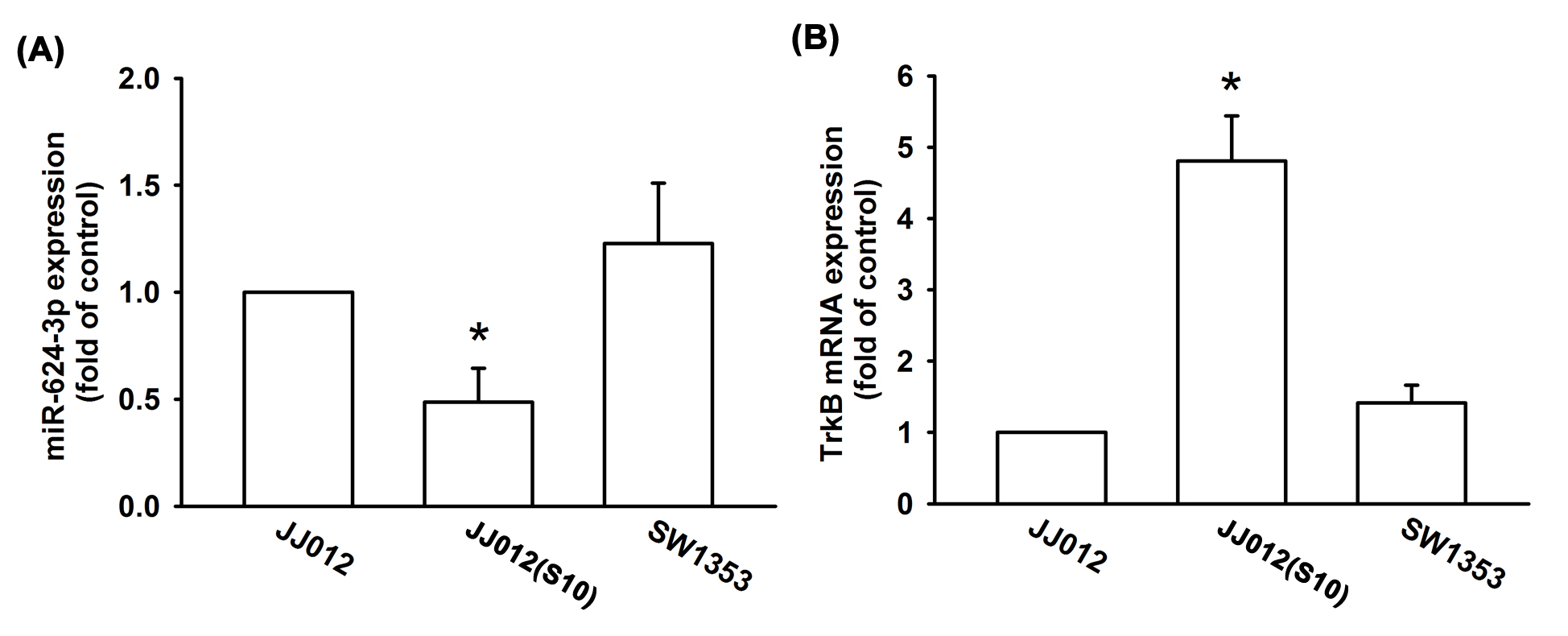


**Fig. S4.** **miR-624-3p and TrkB expression in human chondrosarcoma cell lines.** miR-624-3p (A) and TrkB (B) mRNA expression were measured by RT-qPCR. Quantitative results are expressed as the mean ± SEM. **P* < 0.05 as compared with the JJ012 group.
